# Effects of microstructure on compressive strength of silica sand-enhanced oil well cement at a wide temperature range

**DOI:** 10.1098/rsos.220150

**Published:** 2022-08-10

**Authors:** Zhonggao Chen, Jiapei Du, Annan Zhou, Chunyu Wang, Yuhuan Bu, Huajie Liu

**Affiliations:** ^1^ School of Architecture, Yantai University, Yantai, Shandong 264005, People's Republic of China; ^2^ School of Engineering, Royal Melbourne Institute of Technology, Melbourne, Victoria 3001, Australia; ^3^ College of Material Science and Engineering, Nanjing Tech University, Nanjing 211816, People's Republic of China; ^4^ College of Petroleum Engineering, China University of Petroleum (East China), Qingdao, Shandong 266580, Australia

**Keywords:** fractal dimension, compressive strength, silica sand-enhanced cement, temperature, curing time, pore size distribution

## Abstract

The influence of microstructure of silica-enhanced cement on the mechanical performance of cement is difficult to describe. In this study, we used the scanning electron microscope and image processing method to investigate the relationship between the complicity of cement microstructure and compressive strength under various temperatures and curing times. Fractal dimension was applied to describe the complicity of silica-enhanced cement. The relationships among compressive strength, fractal dimension, temperature, curing time and pore structure of cement were identified. The results show that curing time directly controls the complicity of microstructure of silica-enhanced cement and compressive strength by altering the pore orientation and macropore ratio in silica-enhanced cement. The curing temperature affects the complicity of cement microstructure and compressive strength indirectly by changing the ratio of micropore and small pore. The fractal dimension of silica-enhanced cement shows good correlation with compressive strength. Pore size distribution is the most important factor that influences the complicity of cement matrix and compressive strength of silica-enhanced cement. When building up the macroscopic mechanical performance model of silica-enhanced cement, we should consider the influence of pore size distribution in cement under different curing temperatures and times on the complicity of cement microstructure.

## 1. Introduction

Silica sand has been widely used to enhance the mechanical performance of oil well cement [1–3]. During the utilization of silica sand-enhanced (SSE) cement, the SSE cement will be subject to various temperature (up to 380°C) and curing time conditions [4]. The performance of SSE cement is strongly influenced by curing temperature and curing time [5–16]. For example, Lura *et al*. [7] indicated that higher temperature causes faster shrinkage and the development of self-induced stress. Danish & Mosaberpanah [8] illustrated that high-temperature curing is effective for the properties of cement, such as compressive strength, flexural strength and dry shrinkage. Moreover, plenty of studies investigated the effect of curing time on the performance of cement, which indicated the curing time truly influences the compressive strength, shrinkage and flexural strength [9–12].

The macroscopic performance of cement is strongly related to the variation of microstructure pore volume and pore size distribution of cement. For the microscopic performance, Helmi *et al*. [13] investigated the effects of high-temperature curing on the formation of concrete microstructure, which showed that heat treatment accelerated the propagation of microcracks and increased the capillary pore volume. Patel *et al*. [14] found that temperatures above 46°C have a significant influence on the development of hydrates with microcracks and pores, corresponding with a coarse microstructure. Rong *et al*. [15] indicated that, as the curing time increases, the porosity reduces and the interface between different mineral compositions in cement strengthens. Elsharief *et al*. [16] studied the influence of curing time on the microstructure of the interfacial transition zone and pointed out that long-time curing reduces the high porosity of cement induced by unhydrated cement grain. Cement matrix is a kind of porous media that consists of many pores with different sizes [17,18]. The pore size and pore area distributions in cement matrix strongly influence the mechanical performance of cement [19,20]. The failure and damage of porous materials are generally starting from the weak area, which is more porous when subjected to the external environment variation [21,22]. Therefore, the proper description of microstructure alteration is vital for the understanding of the mechanical performance of silica enhanced cement. Because cement matrix is highly complex, the variation of microstructure under the effect of temperature and curing time is difficult to describe.

The fractal theory has drawn lots of attention to characterize the complexity of porous media in the past decades [23–30]. Gao *et al*. [31] examined the multifractal property of microstructure in cement paste by using X-ray computed tomography to acquire the realistic three-dimensional microstructure. Li *et al*. [32] found that the fractal dimension is an effective approach to evaluate the variation of pore structure at high temperature. Tang *et al*. [33] investigated the pore structure evolution and heat release of cement by utilizing fractal analysis. Wang *et al*. [34] studied the influence of fineness and content on the cement hydration, permeability, mechanical strength and fractal dimension of concrete. Zeng *et al*. [35] performed the fractal analysis of stress-dependent diffusivity of porous cementitious materials, and the pore structure of porous cementitious materials was conducted. Liu *et al*. [36] analysed the variation of pore area and fractal dimension by using a program, which was named as pores and cracks analysis system (PCAS). This program has already been proved to be an effective method to analyse the fractal dimension. However, the relationship among compressive strength, temperature, curing time and pore structure evolution of SSE cement matrix is seldom referred to.

In this paper, fractal analysis is performed on the evolution of pore structure. SSE cement pastes are prepared and subjected to different temperatures and curing times to evaluate the effects of temperature and curing time on the fractal dimension, pore structure and compressive strength. Scanning electron microscope (SEM) images are taken from different samples to carry out the PCAS analysis. The Pearson's coefficient analysis is used to identify the relationship among compressive strength, temperature, curing time, pore volume, fractal dimension and directional probability entropy. The relationship between microstructure of SSE cement and compressive strength was identified for the first time. This study is beneficial for the understanding of the effect of temperature and curing time on the microstructure complexity and compressive strength of SSE cement, and thereby can be the initial step when building the macroscopic performance model of SSE cement.

## 2. Material and methods

### 2.1. Specimen preparation

**Figure 1.**
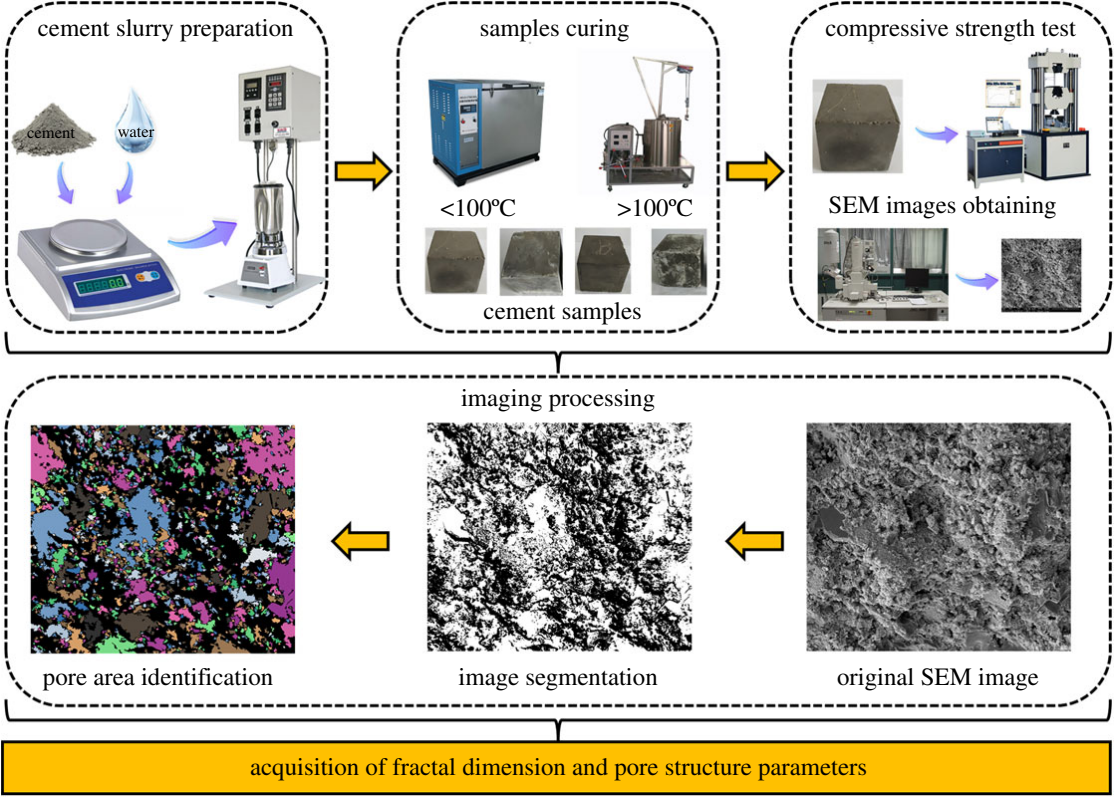
Acquisition of compressive strength and pore microstructure parameters of cement.

The class G oil well cement, which was provided by Sichuan Jiahua Cement Corporation Ltd (Leshan, China), was used in this study. Silica sand was used to enhance the high-temperature resistance property of cement. The chemical composition of cement and silica sand is shown in table 1. The substituting proportion of silica sand is 35% by weight of cement. The water to cement ratio was 0.44 for all the samples. After preparation, as shown in figure 1, the SSE cement slurries were moulded into 5 cm cubes and cured for different curing times, i.e. from 2 to 240 h, at a wide temperature range (from 50 to 380°C). For temperatures less than 100°C, the SSE cement samples were cured in a water bath chamber. For temperatures greater than 100°C, the samples were cured in a high-temperature high-pressure kettle.

**Table 1.** Chemical composition of class G cement, wt%. LOI, Loss on ignition.

CaO	SiO_2_	SO_3_	Fe_2_O_3_	Al_2_O_3_	MgO	Na_2_O	K_2_O	LOI
64.20	19.40	2.80	5.50	4.50	2.00	0.10	0.60	0.9
0.02	98.7	/	0.01	0.01	/	/	/	1.26

### 2.2. Compressive strength evaluation

The compressive strength of SSE cement stone was evaluated at different temperatures (e.g. from 50°C to 380°C) and curing times (e.g. 2 d, 3 d and 10 d). Note that for oil well cement, according to SY/T 6544-2010 (performance requirements for oil well cement slurries), we are mainly concerned about the short-term strength of SSE cement. The 2-day and 3-day compressive strength should be greater than 14 MPa. Meanwhile, the compressive strength for cement cured longer than 7 days should not show any retrogression. Therefore, we chose 2, 3 and 10 days to evaluate the compressive strength of SSE cement. After curing for the designed curing time, the SSE cement stones were extracted from the moulds. The compressive strength of SSE cement stone was evaluated by employing a WEW-300B mechanical performance testing instrument. The compressive strength was carried out based on API RP 10B-3. Each test was performed three times to get static data by calculating the average value.

### 2.3. Microstructure characterization by scanning electron microscopy

When the SSE cement samples were cured for the designed periods, the microstructure of the samples was observed by a ZEISS-SUPRA55 microscope (figure 1). All the observation positions of the samples were selected from the centre of the SSE cement stone to ensure the microstructure information as its original state. By using an ion sputtering equipment, the samples were gold plated to obtain a clear observation. SEM images were taken at different magnifications, and PCAS program was used to obtain the pore area information of the images.

### 2.4. Determination of pore area

**Figure 2.**
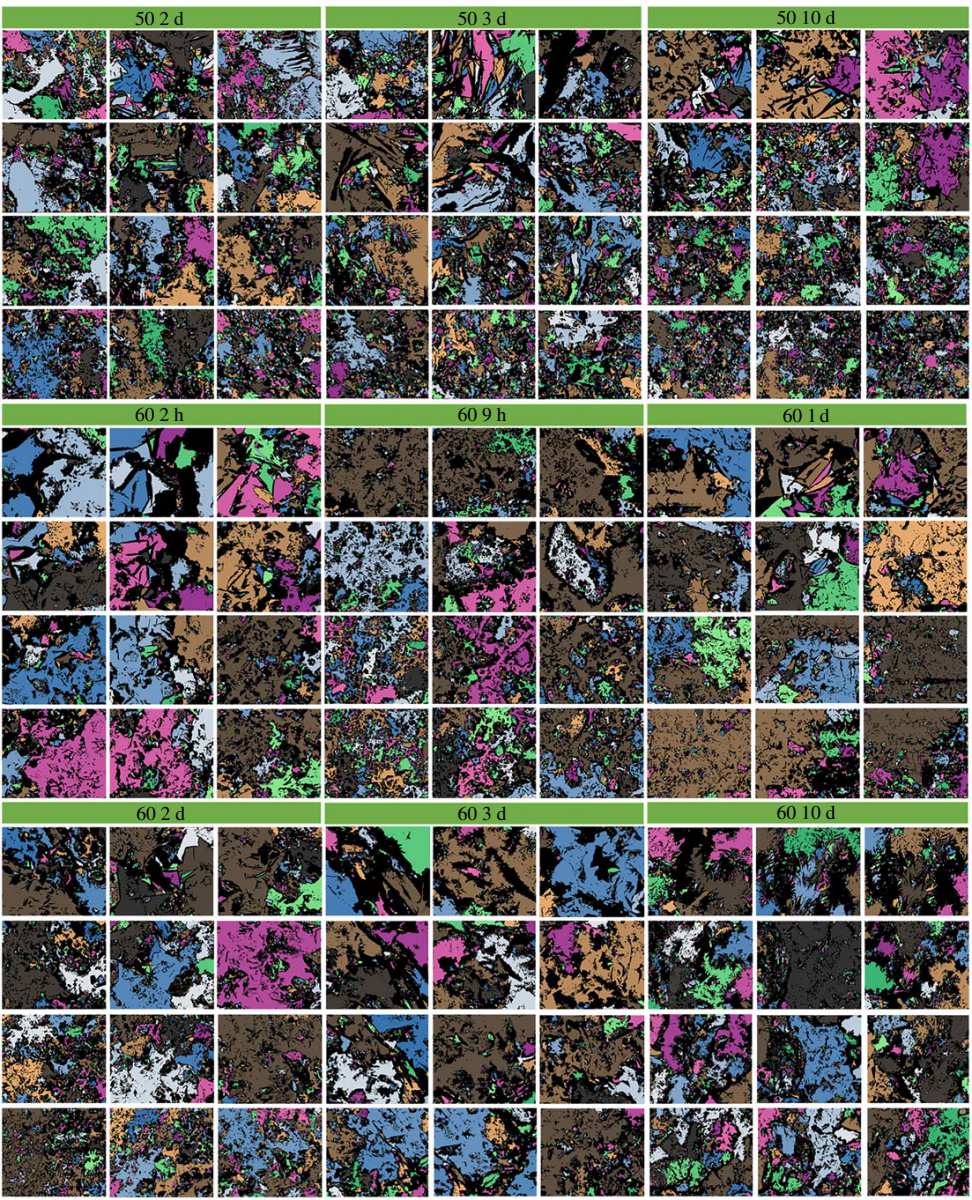
The PCAS-treated SEM images with different magnifications. The samples were cured for 2 d, 3 d and 10 d, respectively, at 50°C. The samples were cured for 2, 9 h, 1 d, 2 d, 3 d and 10 d, respectively, at 60°C.

**Figure 3.**
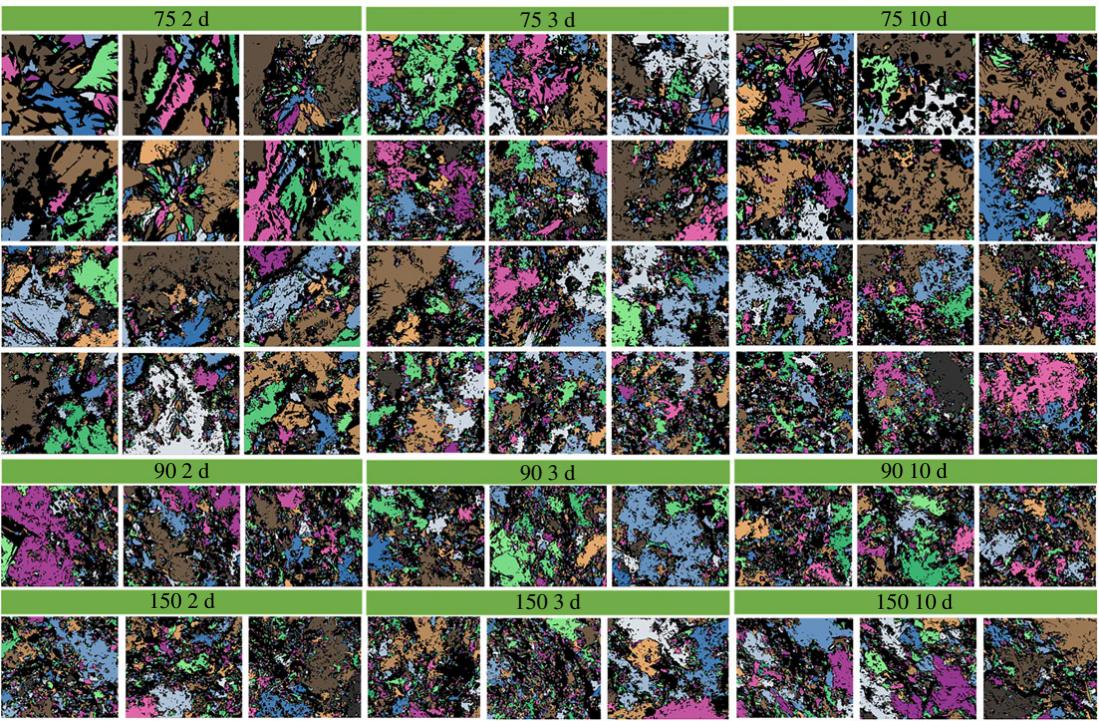
The PCAS-treated SEM images with different magnifications. The samples were cured for 2 d, 3 d and 10 d at 75°C, 90°C and 150°C, respectively.

**Figure 4.**
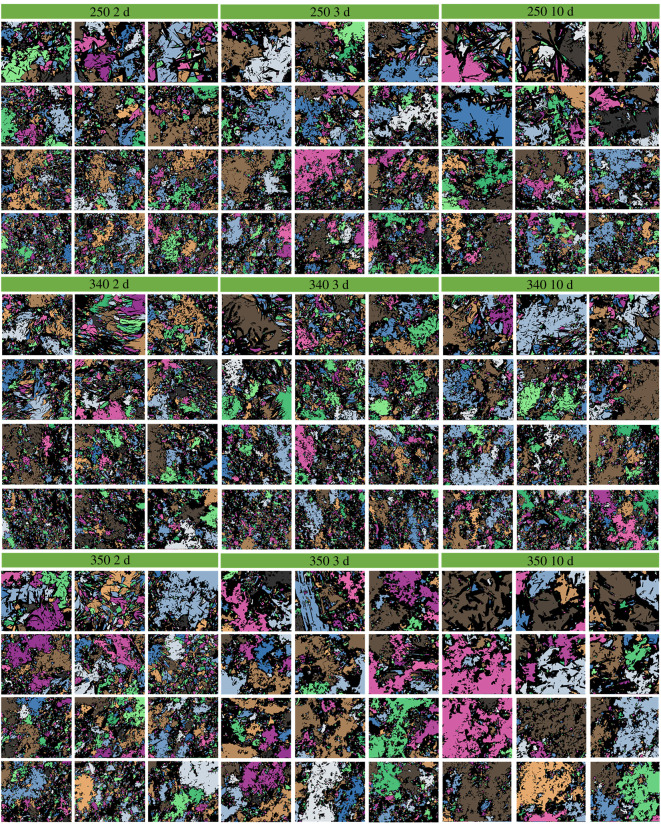
The PCAS-treated SEM images with different magnifications. The samples were cured for 2 d, 3 d and 10 d at 250°C, 340°C and 350°C, respectively.

The PCAS program has already been proved to be a high accuracy and simplicity method to study the cracks and micropores in rock, soil and cementitious materials [36,37]. During the imaging processing, a digital image can be considered as a matrix of pixels, which consists of small squares and dots. The pore areas larger than 20 pixels were identified and evaluated. Figure 1 provides an example of the image processing. The analysis procedure generally involved three steps: (i) the contrasting of image was enhanced to make the boundary between particles and pores clear, (ii) grayscale treatment to segment the pore area and particles through black and white colours, respectively, and (iii) the pore areas were identified and divided into different sizes, which were filled with distinct colours. Therefore, the visualization of pore structure can be realized. It shall be noted that, in the SEM images, specific colour was used for each pore area, i.e. one pore area corresponding to one colour. The black colour represents the solid skeleton of SSE cement. Because the pore area/size distribution varies in different SEM images, no unified standard can be used to label each colour for different SEM images. In other words, if the SEM image is changed, the criteria to label the colour will be changed. Therefore, we did not give the colour for each pore area. The colours were just employed to visualize the pore area in each SEM image. All the PCAS-treated SEM images with different magnifications are shown in figures 2–4. The pore sizes are classified as four levels, which are micropore (areas less than 2 µm^2^), small pore (2–8 µm^2^), mesopore (8–32 µm^2^) and macropore (dimeters larger than 32 µm^2^). Note that the cement matrix is a heterogeneous medium. For example, some positions in 10-day cured samples might be looser than 2-day cured samples. Therefore, we selected 12 SEM images for each sample and calculated the average value of the microstructure parameters to eliminate the influence of heterogeneity.

### 2.5. Determination of fractal dimension and probability entropy

After the PCAS treatment, the fractal dimension *D*_f_ of the selected SEM image can be identified by using the PCAS. When calculating the *D*_f_ in PCAS, *D*_f_ is related with a form factor *ff* by

2.1 $$\log(\;ff)=(1-D_{f})\cdot \log(S)-2c_{1}+\log(4\pi ),$$

where *S* is the total area of the image (unit: pixel); *c*_1_ is a constant.

The probability entropy, which is an indicator for the directionality characteristics of pores [38], was used to study the directional microstructure of the SSE cement. The directional probability entropy (*H*_m_) can be expressed as

2.2 $$H_{m}=-\overset{n}{\underset{i=1}{\sum }}P_{i}\log_{n}P_{i},$$

where *P_i_* is the probability that the pore area occurs within a specific range of angle; *n* represents the number of areas divided by angles, which is equally divided. It shall be noted that the *H*_m_ value is ranging from 0 to 1, and as the *H*_m_ value increases, the pore structure becomes less ordered.

## 3. Results

### 3.1. Compressive strength

**Figure 5.**
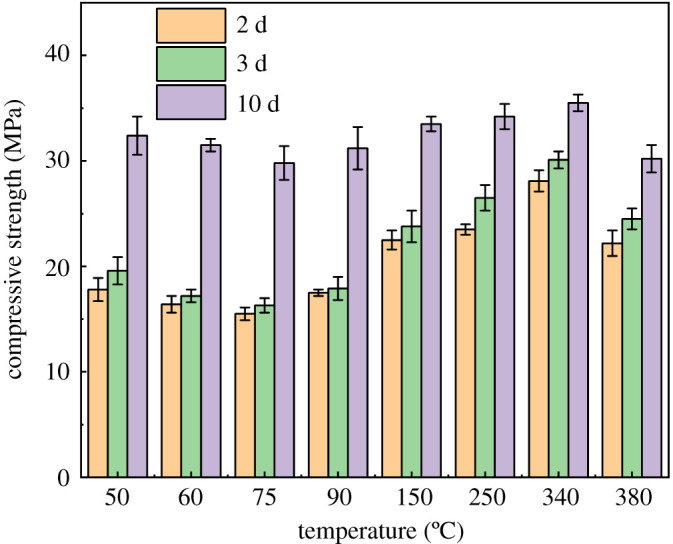
Relationship between compressive strength and temperature at different curing times.

The relationship between compressive strength and temperature at different curing times is shown in figure 5. As we can see, with the increase of temperature, the compressive strength of SSE cement first decreases from 50°C to 75°C and then increases from 90°C to 340°C. After the temperature reaches 380°C, the compressive strength decreases again. With the increase of curing time, the compressive strength increases even at different curing temperatures. Therefore, curing temperature and curing time strongly affect the compressive strength of SSE cement. To further identify the relationship between compressive strength and microstructure of SSE cement, the fractal dimension and pore structure of SSE cement were evaluated in the following sections.

### 3.2. Fractal dimension

Fractal theory is well known for its scale invariance, which means that there is no variation of the original shape regardless of the fractal pattern and scale factor being applied. Thus, at different zoom levels, no discernable difference can be observed in the shape that is presented. Because of the similarity of the fractal medium, it is possible to generate a repetitive medium without a visible change. It has been shown that, in cement matrix, the size–number distribution of pores follows the fractal law [31,32]. The pore areas in cement can be decomposed into one or several representative units with linear size *L* from which the fractal pattern starts. These units possess the same physical properties, such as pore structure, porosity and pore size distribution, but only have one pore with the largest size of *λ*_max_. Therefore, the number of pore areas with diameter *λ* can be written as

3.1 $$N(\lambda )={\left(\frac{\lambda }{\lambda_{\max}}\right)}^{-D_{f}}.$$

In the meantime, based on the classical coastline-divider rule method [39], the number of pore areas with the effective cross-section area *A*(*λ*) yields a power-law equation

3.2 $$N(A(\lambda ))={\left(\frac{A(\lambda )}{A_{\max}}\right)}^{-D_{s}/2},$$

where *D*_s_ is the fractal dimension of pore area. For a specific pore space consisting of straight flow path with diameters fractally distributed, the *N*(*λ*) = *N*(*A*(*λ*)) is valid and therefore *D*_f_ = *D*_s_ is satisfied, which means the *D*_f_ is also the pore size or pore area fractal dimension of pore area with fractal characteristic.

#### 3.2.1. Effect of scale effect on fractal dimension

**Figure 6.**
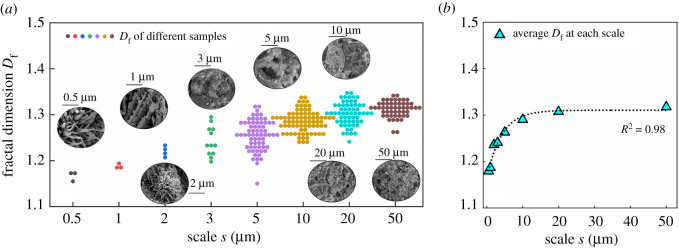
Effects of magnification on the fractal dimension. (*a*) Fractal dimension of different samples at different magnification; (*b*) relationship between fractal dimension and scale factor *s*.

Sun *et al*. [40] indicated that the magnification of SEM image shows a considerable scale effect on the fractal dimension. Therefore, the SEM images were taken from different magnifications to investigate the influence of scale on the fractal dimension of cement microstructure. The magnifications of 50 000, 25 000, 13 000, 7000, 5000, 2000 and 1000 are represent by the scale of 0.5, 1, 2, 3, 5, 10, 20 and 50 µm, respectively. Figure 6*a* shows the effects of magnification on the fractal dimension. Because the SEM images are two-dimensional images, the fractal dimension basically varies from 1 to 2. Even though the fractal dimensions show a relatively discrete distribution in each magnification, with the decrease of magnification, i.e. the increase of scale factor *s*, the increase of fractal dimension can be observed. The average fractal dimension at each scale was calculated and collected in figure 6*b*. As we can see, the average fractal dimension displays a rapid increase as the scale increases from 0.5 to 10 µm. After that, from 10 to 50 µm, the average fractal dimension keeps nearly unchanged. A greater magnification possibly contains less information of SSE cement microstructure and thereby leads to a lower fractal dimension. Meanwhile, after the scale factor increases to more than 10 µm, it is enough to allow the image to obtain the whole complexity of the microstructure of SSE cement. This phenomenon leads to the average fractal dimension keeping unchanged when the scale factor is larger than 10 µm. Therefore, the scale factors of 20 and 50 µm were used to evaluate the effect of temperature and curing time on the fractal dimension and pore structure evaluation of SSE cement.

#### 3.2.2. Effect of curing temperature on fractal dimension

**Figure 7.**
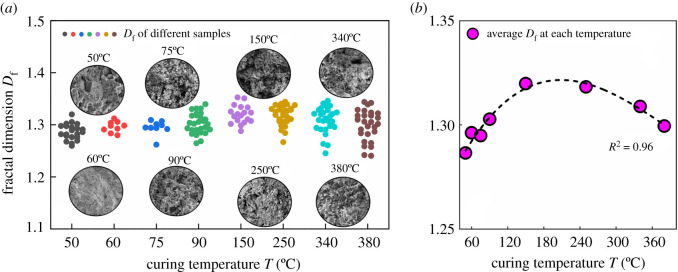
Effects of curing temperature on the fractal dimension. (*a*) Fractal dimension of different samples at different temperatures; (*b*) relationship between fractal dimension and curing temperature *T*.

To investigate the effect of curing temperature on the fractal dimension of SSE cement microstructure, the SSE cement samples were prepared under different curing temperatures. The fractal dimension was calculated under the magnification of 2000 and 1000 to eliminate the scale effect. The relationship between curing temperature and fractal dimension is shown in figure 7. As we can see in figure 7*a*, with the increase of curing temperature, the fractal dimension varies from 1.25 to 1.35. No obvious variation can be observed from figure 7*a*, because the data are quite discrete. After the average fractal dimension was calculated, a clear variation can be observed, in which the fractal dimension first increases and then decreases with the increase of curing temperature. When cured at low temperatures (i.e. 50°C–60°C), a dense structure can be observed from the SEM images in figure 7*a*, and thereby leads to a low fractal dimension. As the temperature increases from 75°C to 250°C, the microstructure of SSE cement becomes loose. The loose structure results in high complexity and a large fractal dimension. After the temperature rises to more than 340°C, the microstructure of SSE cement becomes dense again, and therefore a low fractal dimension can be observed.

#### 3.2.3. Effect of curing time on fractal dimension

**Figure 8.**
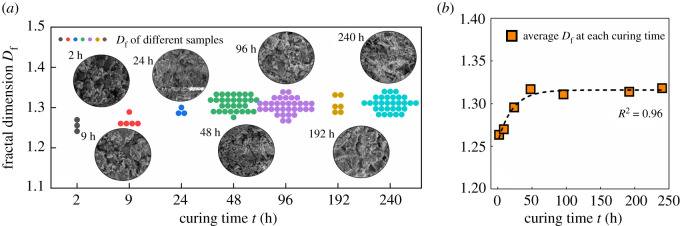
Effects of curing time on the fractal dimension. (*a*) Fractal dimension of different samples at different curing times; (*b*) relationship between fractal dimension and curing time.

To investigate the effect of curing time on the fractal dimension of SSE cement microstructure, the SSE cement samples were prepared under different curing times. The fractal dimension was calculated under the magnification of 2000 and 1000 to eliminate the scale effect. The relationship between curing time and fractal dimension is shown in figure 8. With the increase of curing time, the average fractal dimension first increases and then maintains a stable state. As we can see from figure 8*a*, the microstructure becomes denser when the curing time increases from 2 h to 48 h, and thereby induces a rapid increase of fractal dimension. After 48 h curing, the microstructure of SSE cement shows no significant alteration as the curing time increases from 48 h to 240 h. Therefore, the average fractal dimension displays a stable value after 48 h curing.

### 3.3. Pore structure evolution

#### 3.3.1. Pore area analysis

**Figure 9.**
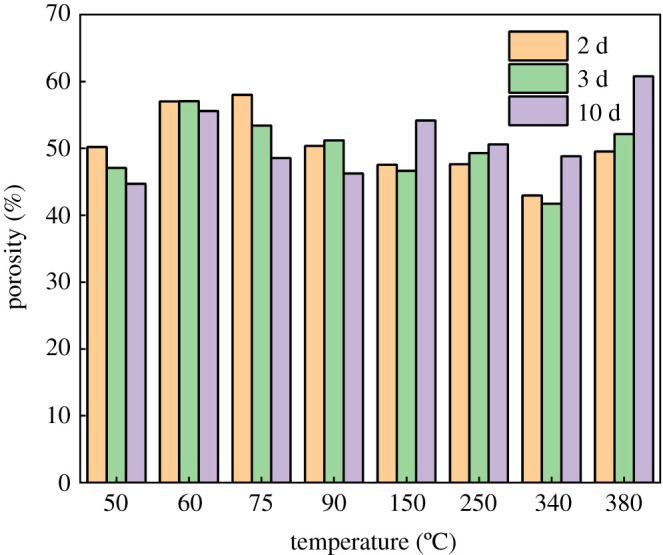
Relationship between temperature and porosity at different curing times.

The rationality of using SEM analysis to determine the porosity has already been proved by previous studies [38,41,42]. Therefore, SEM analysis was employed here to investigate the pore area variation of SSE cement under different temperatures and curing times, as shown in figure 9. When the temperature rises from 50°C to 75°C, the 2-day porosity increases. Meanwhile, at this temperature interval, with the increase of curing time (i.e. from 2 d to 10 d), the porosity decreases. This is because part of β-C_2_S is transferred to portlandite when the temperature increase from 50°C to 75°C [5]. The transition of mineral composition and crystalline results in a large quantity of pores and cracks, as well as high porosity [5]. When the temperature varies from 90°C to 340°C, the 2-day porosity decreases. However, at 150°C, 250°C and 340°C, the porosity increases with the increase of curing time. This is due to long-term curing of SSE cement at high temperatures leading to the dehydration of hydration products [6], and thereby enlarging the void space in SSE cement. At 380°C, an obvious rise of porosity can be observed in figure 9. These phenomena illustrate that the curing time and curing temperature can not only influence the fractal dimension, but also the pore area variation of cement.

#### 3.3.2. Pore size analysis

**Figure 10.**
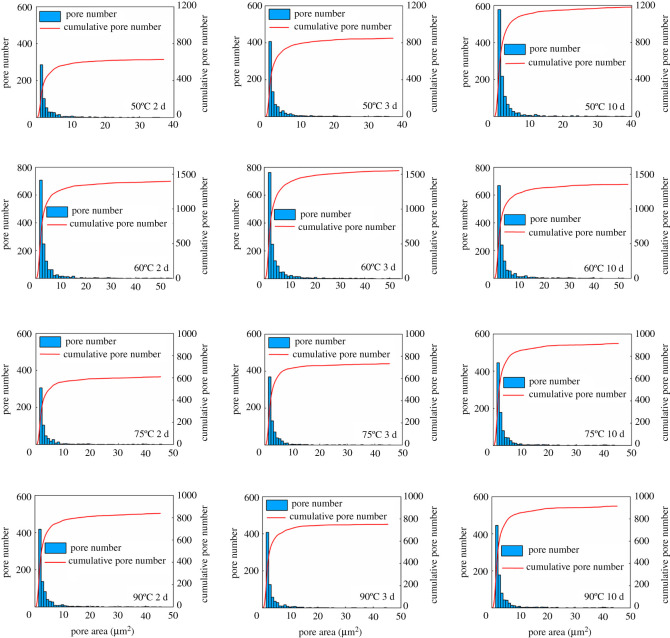
Microstructure pore size distribution of SSE cement at various curing temperatures and curing times (temperature ranging from 50°C to 90°C).

**Figure 11.**
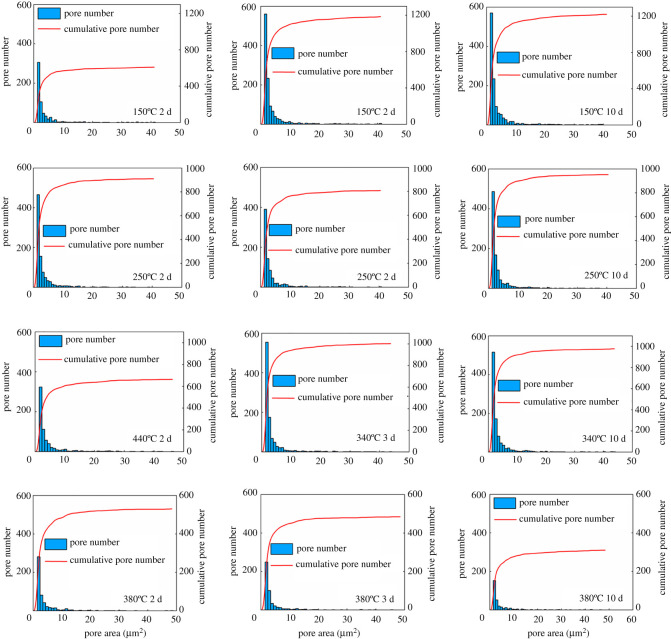
Microstructure pore size distribution of SSE cement at various curing temperatures and curing times (temperature ranging from 150°C to 380°C).

The pore size distribution in the cement matrix is another vital factor influencing the complicity of cement microstructure and the corresponding mechanical properties [43,44]. To further investigate the effects of temperature and curing time on the pore size distribution, the microstructure pore size distributions at various temperatures and curing times are provided in figures 10 and 11. From figure 10, at 50°C, with the increase of curing time, the quantity of pore number rises, even though the porosity of SSE cement decreases. The increase of pore number is mainly due to the increase of micropore (less than 2 µm^2^) numbers. When the temperature reaches 60°C, the overall quantity of pore number at all curing times is greater than that at 50°C, which shows a good agreement with the porosity results. At 75°C and 90°C, the overall quantity of pore number, as well as the micropore number are lower than that at 60°C, which illustrates that curing at a relatively high temperature (i.e. 75°C and 90°C) leads to a reduction of micropores quantities.

When cured at 150°C, as shown in figure 11, the micropore quantity and overall pore quantity both increase with the curing time. After the temperature reaches 240°C and 340°C, micropore quantity displays a little lower value than that at 150°C, which indicates good agreement with the porosity results. At 380°C, the micropore quantity shows an obvious decrease, while the porosity increases (figure 9). This is due to, when curing at 380°C, the quantity of pore areas which is greater than 50 µm^2^ increases significantly, and the quantity of micropores reduces. In addition, with the increase of curing time, the quantity of micropores decreases as well. Therefore, according to the analysis above, the curing temperature and curing time can truly affect the pore size distribution of the SSE cement matrix.

#### 3.3.3. Pore orientation analysis

**Figure 12.**
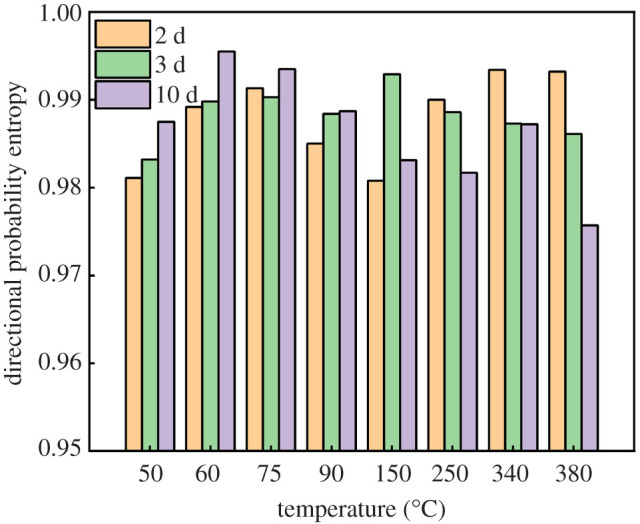
Relationship between directional probability entropy and temperature at different curing times.

The pore directional probability entropy is another key parameter to reflect the complicity of the SSE cement microstructure [38]. Figure 12 shows the relationship between directional probability entropy and temperature at different curing times. When the temperature varies from 50°C to 90°C, the probability entropy increases with the rise of curing time. When the temperature rises to higher than 150°C, the probability entropy decreases with the increase of curing time. Temperature does not show an obvious influence on the probability entropy. Therefore, different from porosity and pore size distribution, the pore orientation is mainly influenced by curing time, rather than curing temperature.

## 4. Discussion

**Figure 13.**
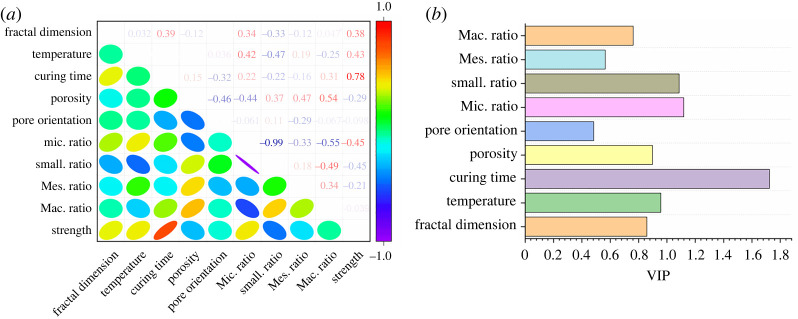
Mechanism illustration. (*a*) Pearson's correlation coefficient matrix of different factors. (*b*) Variable importance in projection.

As discussed in the previous sections, the curing temperature and curing time can influence the compressive strength, fractal dimension, porosity, pore size distribution and pore orientation. Even though the relationship among porosity, compressive strength and curing temperature is well known, the relationship among the microstructure factors, curing temperature and curing time, as well as its influence on compressive strength is still unclear. Therefore, our study mainly focused on the effects of curing temperature and curing time on multi-microstructure parameters, such as proportion of pores at different levels (i.e. micropore, small pore, mesopore and macropore), pore orientation and fractal dimension. The relationship between the microstructure parameter and compressive strength was identified. Pearson's coefficient and variable importance in projection (VIP) analysis were applied to identify the relationships. Figure 13*a* shows the Pearson's correlation coefficient matrix of different factors. The scale bar represents the Pearson's coefficient value (*r*), where the coefficient ranging from −0.5 to −0.3 and 0.3 to 0.5 represent medium correlation, as well as −1.0 to −0.5 and 0.5 to 1.0 mean strong correlation [45]. The Pearson's coefficient values are shown in the upper portion of the matrix. The shape of ellipse reflects the magnitude of the correlation. For example, when the shape of ellipse is more like a circle, the correlation is weak with a low correlation coefficient. In figure 13*a*, the pore size in SSE cement was classified as micropore, small pore, mesopore and macropore, and the ratio of these pores in total pore area was calculated. Figure 13*b* shows the VIP results, which represent the variable importance of each parameter on the fractal dimension of SSE cement. A greater variable importance value means the parameter is more important to the variation of fractal dimension.

### 4.1. Effect of curing temperature

As shown in figure 13*a*, the temperature shows a positive correlation with the micropore ratio (*r* = 0.42). In the meantime, the small pore ratio shows a negative correlation with temperature (*r* = −0.47). The porosity, pore orientation, mesopore ratio and macropore ratio display weak correlations with temperature. For the compressive strength, it shows medium correlation with temperature (*r* = 0.43). Similar to temperature–micropore ratio and temperature–small pore ratio correlation, the compressive strength also displays medium correlation with micropore and small pore ratio. This result illustrates that the temperature alters the compressive strength of SSE cement by changing the quantity of micropores and small pores.

Generally, for ordinary Portland cement, with the increase of temperature, a decrease of compressive strength occurs due to the phase change in cement matrix. Taylor [46] indicated that if the temperature within the cement exceeds 70°C, serious loss of durability can occur because of the formation of ettringite increased the microcracking. When the temperature reaches 110°C, part of the chain-like or network calcium silicate hydrates (CSH) transfers to plate-like Ca_2_SiO_4_ ⋅ H_2_O (C_2_SH) [47], which leads to the reduction of mechanical performance of cement [48]. When the temperature increases to 200°C, nearly all the CSH phases in cement transfer to the C_2_SH [49]. After incorporating the silica sand, when temperature goes above 110°C, the tobermorite and xonotlite form in the SSE cement, which improve the mechanical property and durability of SSE cement [50]. After the temperature rises to about 400°C, the decomposition of calcium hydroxide reduces the compressive strength again. This might be the reason for the distinct temperature–strength relationship observed in this study (figure 5). The hydration phase variation influences the quantity of micropores and small pores, and thereby affects the compressive strength of SSE cement.

### 4.2. Effect of curing time

The curing time shows a medium correlation with pore orientation and macropore ratio (figure 13*a*). A longer curing time leads to a more complicated pore orientation (*r* = −0.32) and a greater macropore ratio (*r* = 0.31). The curing time displays a weak influence on porosity, micropore, small pore and mesopore ratio. Meanwhile, the compressive strength shows strong correlation with curing time (*r* = 0.78), which indicates that the curing time affects the compressive strength of SSE cement by controlling the pore orientation and macropore ratio in SSE cement. As also confirmed by Pang *et al*. [6], long-term curing (up to 142 days) of silica-enriched cement experiences deterioration in mechanical and physical performance, such as permeability and compressive strength. Wang *et al*. [50] studied the 28 days strength retrogression of nanosilica-enhanced cement under high temperature. The strength of nanosilica-enhanced cement increases as the rise of curing time. The differences might be caused by the distinction of silica dosage, particle size, curing temperature and curing time. Even though different results can be observed from different studies, the results all show that curing time has great influence on the compressive strength of SSE cement. The pore orientation and macropore ratio might be the main reasons that led to the changing of permeability and compressive strength of SSE cement in terms of curing time.

### 4.3. Effect of fractal dimension

The influence of curing temperature on fractal dimension is limited (*r* = 0.03). However, the curing time shows a considerable effect on fractal dimension (*r* = 0.39). Meanwhile, the quantity of micropore and small pore also influence the fractal dimension with a Pearson's *r* of 0.34 and −0.33, respectively. Even though the porosity of SSE cement is well controlled by all the pore property parameters, i.e. the *r* value is −0.46 for pore orientation, −0.44 for micropore ratio, 0.37 for small pore ratio, 0.47 for mesopore ratio and 0.54 for macropore ratio, it displays weak correlation with the fractal dimension. Therefore, the curing time of SSE cement can directly control the complicity of cement microstructure by altering the pore orientation and macropore ratio in SSE cement. The curing temperature influences the complicity of SSE cement microstructure indirectly by changing the micropore and small pore ratio. As also confirmed by Li *et al*. [32], temperature can truly influence the fractal dimension by altering the porosity of cement. High temperature, long-term curing and a large quantity of micropore results in a high complicity (i.e. great fractal dimension) microstructure of SSE cement. A high complicity of microstructure of SSE cement also leads to a great compressive strength. As also can be confirmed by the VIP analysis in figure 13*b*, micropore and small pore ratio and curing time are three important parameters that influence the variation of compressive strength of SSE cement. The changing of pore orientation shows a weak contribution to the compressive strength. In a word, pore size distribution is the most important factor that influences the complicity of the SSE cement matrix, as well as compressive strength. When building up the macroscopic performance model of SSE cement, we should consider the influence of pore size distribution in SSE cement under different curing temperatures and times on the complicity of cement microstructure, and thereby designing the mechanical performance of SSE cement.

## 5. Conclusion

In this study, the influences of curing temperature and curing time on the complicity of silica sand-enhanced cement microstructure and compressive strength were investigated by the SEM analysis and imaging processing approach. Based on the experimental results and statistical analysis, the following conclusions can be drawn.

(1) The magnification of SEM image shows a considerable scale effect on the fractal dimension. When using the image processing method to investigate the fractal dimension, proper magnification of images should be selected to eliminate the scale effect.
(2) Curing temperature influences the microstructure and compressive strength of silica sand-enhanced cement by changing the quantity of micropores and small pores. The correlations among curing temperature, porosity, pore orientation, mesopore ratio and macropore ratio are weak.
(3) Curing time displays a weak influence on porosity, micropore, small pore and mesopore ratio, while affecting the microstructure and compressive strength of silica sand-enhanced cement by controlling the pore orientation and macropore ratio in cement.
(4) High temperature, long-term curing and a large quantity of micropore result in a high complicity microstructure and a great compressive strength of silica sand-enhanced cement. The changing of pore orientation shows a weak contribution to the compressive strength.
(5) The compressive strength of silica sand-enhanced cement shows good correlation with the complexity of microstructure of silica sand-enhanced cement. Pore size distribution is the most important factor that influences the complicity of the silica sand-enhanced cement matrix, and thereby affects the mechanical performance of silica sand-enhanced cement.

## Data Availability

The data can be accessed through a link to the Dryad Digital Repository https://doi.org/10.5061/dryad.b5mkkwhfg [51].
